# Combinational Periprosthetic Hip Joint Infection and Fracture: A Two-Stage Revision with Long Femoral Stem Spacers

**DOI:** 10.5704/MOJ.2503.005

**Published:** 2025-03

**Authors:** TV Duong, TB Duong, DT Tu, NQT Quyen, H Tan, TNK Hung

**Affiliations:** 1 Department of Cosmetic Surgery, Cho Ray Hospital, Ho Chi Minh City, Vietnam; 2 Department of Orthopaedics, Cho Ray Hospital, Ho Chi Minh City, Vietnam; 3 Department of Orthopaedics, Tam Anh Hospital, Ho Chi Minh City, Vietnam; 4 Faculty of Pharmacy, Nguyen Tat Thanh University, Ho Chi Minh City, Vietnam

**Keywords:** periprosthetic hip infections, periprosthetic hip fractures, long femoral stem spacer, two-stage revision, combinational periprosthetic hip joint infection and fracture

## Abstract

**Introduction:**

Periprosthetic joint infection combined with periprosthetic fracture rarely occurs simultaneously. Once all components of the periprosthetic joint infection were removed, antibiotic spacers were placed. Moreover, periprosthetic fractures require fixing. We use a long femoral stem spacer molded intra-operatively via a self-design metal mold as a novel treatment method for the periprosthetic fracture combined with infection.

**Material and Methods:**

The study reviewed 12 patients who underwent two-stage revision arthroplasty with long femoral stem spacers over 6 years. During a minimum of two years of follow-up, the outcomes were evaluated, including reinfection rates, reimplantation rates, and re-operation rates, as well as the success rate based on the MSIS criteria.

**Results:**

Twelve patients underwent two-stage revision with a long femoral stem spacer between stages. A mean follow-up period of 9.58 months followed infection (range 2 to 28 months). In 11 patients (91.67%), the infection was eradicated. There was one patient (8.33%) who required a second 2-stage revision and subsequently cleared their infection. The long femoral stem spacer was repeated in three patients (25%). After eradicating the infection, 9 patients (75%) underwent 2nd stage revision, on average 8.56 months after the first stage. At an average of 27.92 months (range 8 - 65 months) post-operatively, three (25%) long femoral stem spacers remained in place.

**Conclusion:**

Using long femoral stem spacers, both periprosthetic joint infections and periprosthetic fractures can be treated simultaneously. In the cases with multiple organisms, we mixed one pack of bone cement with 2g of Vancomycin and 2g of Meropenem, resulting in satisfactory results.

## Introduction

There are many serious complications associated with arthroplasty, including periprosthetic joint infections (PJI) and periprosthetic fractures (PPF). Both of these conditions require a structured and complex approach in terms of their treatment^1-3^. When both complications occur simultaneously, one is confronted with a situation that appears as if it is unsolvable^4^. Therefore, there exists an inherent problem with separate and, in some aspects, opposing treatment concepts available for PJI and PPF. PPF requires internal fixation with foreign material for fracture stabilisation, but PJI requires surgical infection eradication along with complete removal of the prosthesis.

A two-stage approach is currently the most effective way to treat periprosthetic infections. In this method, the components are removed and a temporary spacer with antibiotics is inserted^5,6^. It has been reported that approximately 70% to 90% of cure rates are attributable to this approach. The combination of PJI and subsequent PPF has been reported to be exceptionally rare, devastating, and likely to require prolonged antibiotic treatment, as it may necessitate a lengthy course of medication. In addition, it is common that a number of surgical interventions will also be required, resulting in poor outcomes^7-9^. Infection around implants and subsequent fractures are unlikely to occur simultaneously. This can lead to prolonged antibiotic treatment, extensive surgical interventions, and poor outcomes^8-10^. The worst outcome of this situation is that the patient will have to undergo arthrodesis or an amputation. In the event of a bone fracture, an orthopaedic surgeon is responsible not only for eradicating the infection but also for maintaining the limb's functionality and providing a stable fixation so that the bone can begin to heal on its own.

The purpose of this study is to provide definitive treatment for patients with PJI combined with PPF, and we present a novel treatment method in which a long femoral stem spacer is used in order to accomplish this goal. For molding the long femoral stem spacer, we designed the metal mold that is used to mold the long femoral stem spacer intra-operatively, directly after the surgery has been completed.

## Materials and Methods

The purpose of this study was to perform a retrospective analysis based on all data collected on patients who had PJI combined with PPF from 2016 to 2022 at Cho Ray Hospital, Vietnam. In order to conduct the present study, institutional review board (IRB) approval was obtained - ethical code 0713/CN-HĐĐĐ from Cho Ray Hospital. Among the patients we enrolled in this dataset were those with signs of infection based on the MSIS criteria as well as radiograph results confirming PPF, as defined by the Vancouver classification. Patients who had been diagnosed with proximal femoral fractures before the time of arthroplasty were excluded from the study. We also validated all patients who met the criteria for participating in the study using microbiology and infectious diseases databases. Also, standardised questionnaires were used to gather clinical, pathologic, microbiological, and management data, such as the type and date of PJI operations, comorbidities, and outcomes.

**Fig. 1:**
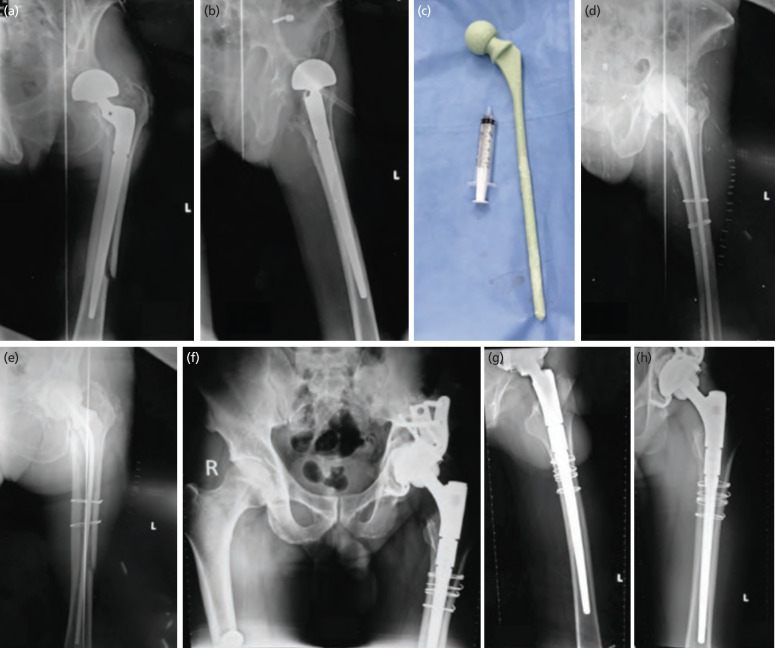
Two-stage revision in a patient who received PJI in combination with PPF. (a, b) A1 and A2 PJI in the combination with PPF. (c) B1 long femoral stem spacer. (d, e) B2 and B3 replaced all the components by long femoral stem spacer. (f – h) C1-C3 two-stage revision with a new prosthesis.

Our hospital had 208 patients with PJI during the study period. Clinical, biological, and microbiological data, comorbidities, and final results of PJI patients were reviewed. To control infection and stabilise fractures after the resection arthroplasty, a long femoral stem spacer with antibiotics was utilised in all patients (over the next 3–6 months). Among the 12 patients with PJI combined with PPF, 3 are awaiting revision arthroplasty (Fig. 1).

**Fig. 2:**
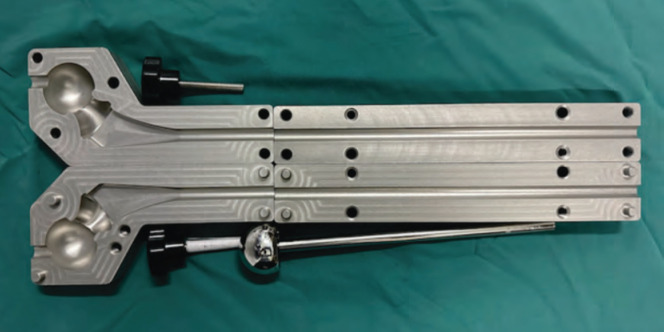
The mold of the long femoral stem spacer.

As described in my study, we have found that the most effective therapies for PJI combined with PPF include debridement, excision of any infected or damaged components, and implanting an antibiotic-impregnated bone cement long femoral stem spacer. The next stage of treatment was arthroplasty once the condition of the PJI was confirmed to be stable, which was shown by the absence of any signs of ongoing infections and the normal levels of erythrocyte sedimentation rate and serum C-reactive protein analysis. After the insertion of a spacer, we recommend that the patient take intravenous antibiotics for two weeks following the insertion of the spacer and take them orally for four to eight weeks afterward to ensure that the infection is controlled. In the case of persistent infection, other surgeries with implantations of long femoral stem spacers impregnated with antibiotics might be required in order to treat this condition (Fig. 2).

**Fig. 3:**
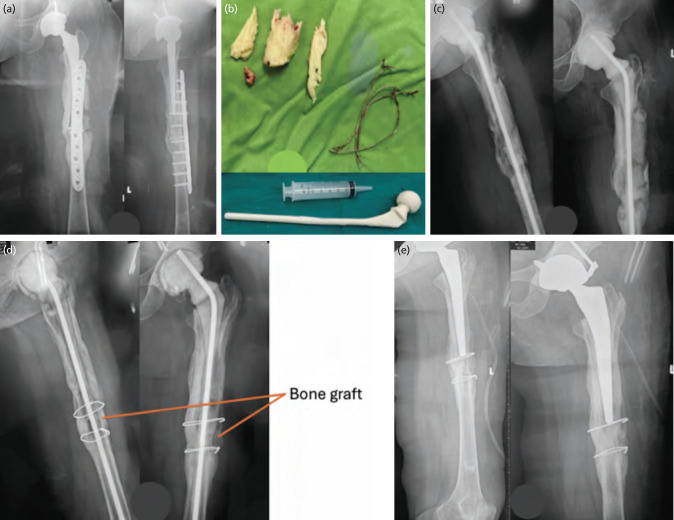
(a) PJI combined with PPF. (b) B1 Removing all components and necrosis bone, (c) B2 replacing the femoral shaft spacer and filling the dead space with cement, (d) B3 removing the cement spacer from the femoral shaft and preparing the bone graft. (e) Replace the long femoral shaft spacer with the new prosthesis.

Design and construction of a custom spacer mold; The mold was fabricated with a computerised High Precision CNC Metal Engraving Machine ST4040M and is comprised of four components. To make the mold, an aluminium alloy called Alloy 5754 is used. This alloy is composed of magnesium in the range of 2.6-3.6%, and it was coated with a ceramic coating to give it a marine-grade standard. Cleaning and sterilising the mold is one of the ways that it can be reused. A long femoral stem spacer was molded intra-operatively with 4g of Vancomycin and/or 4g of Meropenem (Fig. 3). The type of antibiotic varies based on its antibiogram; however, they are all broad-spectrum, heat-stable, and powder-based. Fig. 3 is the mold of the long femoral stem spacer.

## Results

**Fig. 4:**
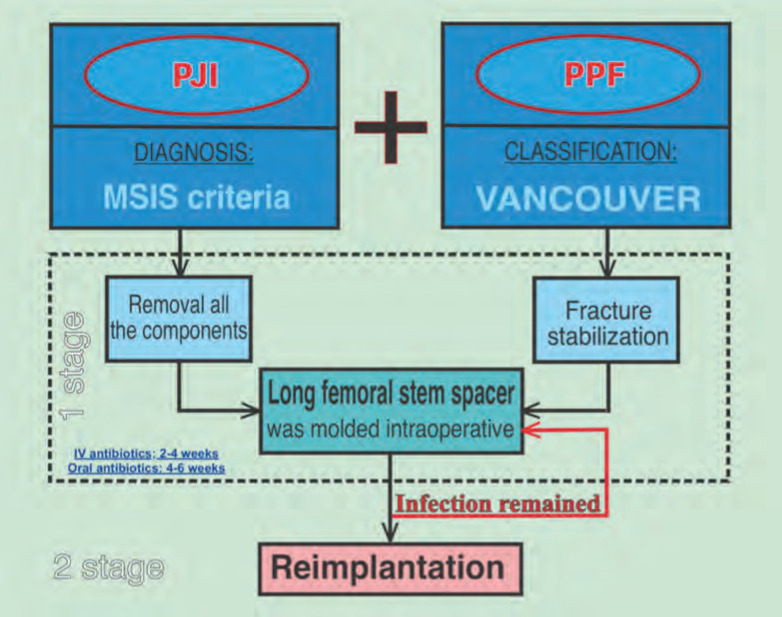
Diagnosis and treatment of PJI combination with PPF.

The research period was characterised by 12 out of 208 (5.77%) PJI cases associated with PJI and PPF. A total of nine males and three females were found in this research. According to Table I, the median age of the patients was 53.75 years (range 38–76 years). The average time between the arthroplasty and the detection of infection was 9.58 months (range 2 to 28 months). There were seven cases of sinus tracts communicating with the joint as well as symptoms of pain associated with the sinus tracts. Synovial culture confirmed the diagnosis in seven cases, with an increase in ESR associated with all positive PCR tests. The present study found that five patients had concomitant infections caused by more than one organism, and eight cases were infected with Staphylococcus aureus, which was accompanied by methicillin-resistant S. aureus. Radiographically, based on the Vancouver classification, there were 2 cases of type A, 3 cases of type B1, 2 cases of type B2, 3 cases of type B3, and 2 cases of type C (Fig. 4).

**Table I:** PJI/PPF clinical characteristics.

Patient No	Year of birth/ gender	Comorbidity	Diagnosis	Organism	Type**	Infected time (months)	Spacer duration (months)	Number of spacer	Revision	Follow-up time (months)	Outcome
01	1985/F	Neck + shaft femoral fractures. Diabetes	ESR, CRP, Sinus track	S. Aureus - MRSA; A. Baumannii, P. Aeruginosa	C	9	16	1	Yes	8	eradicated
02	1966/M	AVN, Flypertension, Diabetes	ESR, CRP, Sinus track Synovial culture	P. Aeruginosa, K. pneumoniae - ESBL, S. Aureus	B3	2	6 (now)*	2	No	23	eradicated
03	1971/M	Bi - AVN	ESR, CRP, Synovial culture	S. Aureus - MRSA	B1	10	14	1	Yes	65	eradicated
04	1969/M	AVN	ESR, CRP, Sinus track	S. Aureus	C	8	3	1	Yes	19	eradicated
05	1979/M	Bi - AVN	ESR, CRP, Synovial culture	S. Aureus, A. baumannii	B2	28	5	1	Yes	11	eradicated
06	1960/F	AVN, Hypertension	ESR, CRP, Sinus track	K. pneumoniae - ESBL, S. Aureus - MRSA	B3	16	7 (now)*	2	No	20	eradicated
07	1975/M	Bi - AVN	ESR, CRP Synovial culture	S. Aureus - MRSA	B1	6	11	1	Yes	17	eradicated
08	1979/M	Gout, Bi - AVN	ESR, CRP, Sinus track	P. Aeruginosa, A. baumannii	B1	7	9	1	Yes	54	eradicated
09	1968/M	AVN, Hypertension, Diabetes	ESR, CRP, Sinus track	S. Aureus - MRSA	A	3	5	1	Yes	22	eradicated
10	1977/F	Bi - AVN	ESR, CRP, Synovial culture	S. Aureus - MRSA	A	4	8	1	Yes	19	eradicated
11	1955/M	Bi - AVN, Hypertension	ESR, CRP, Synovial culture	S. Aureus - MRSA	B2	9	6	1	Yes	17	eradicated
12	1947/M	AVN, Abdominal wall hernia. Psoas abscess	ESR, CRP, Sinus track. Synovial culture	K. pneumoniae - ESBL, S. Aureus - MRSA, A. baumannii	B3	13	6 ( now)*	3	no	60	Bad

• Klebsiella pneumoniae: K. pneumoniae, ESBL: Extended-spectrum β-lactamase, Staphylococcus aureus: S. Aureus, MRSA: Methicillin-resistant Staphylococcus aureus, Acinetobacter Baumannii: A. Baumannii, Pseudomonas aeruginosa: P. Aeruginosa, Avascular necrosis of hip: AVN, Bilateral avascular necrosis of hip: Bi – AVN, ESR: Erythrocyte Sedimentation Rate, CRP: C-reactive protein

• (Now)* Retain the spacer until now, Type**: Vancouver classification

The patients with coinfections received cement spacers that contained 2g of Vancomycin and 2g of Meropenem per 40g of cement. According to their antibiogram, they were prescribed oral antibiotics for coinfections in four to six weeks. It has been reported that all 11 cases have been successfully eradicated from infection after the last follow-up period. Reimplantation of the prosthesis is pending in the two remaining cases. Generally, the duration of the spacer was 8.56 months, except for 3 cases that are still using more than one spacer. Furthermore, nine cases of PJI combined with PPF were confirmed to have been successful in two-stage revision arthroplasty. As a result, 11 of the patients had good outcomes and, on average, the follow-up time was 27.92 months (range 8 - 65 months) after reimplantation.

## Discussion

There is no doubt that two-stage exchange arthroplasty is considered the gold standard for the treatment of chronic PJI^11,12^. A patient with PJI combined with PPF faces a very challenging situation, both from the point of view of the patient and the treating surgeon^13^. It is one of the major goals of the treatment to salvage the limb and eradicate the infection, regain limb function, and maintain joint stability throughout the entire treatment. The greatest challenges are the conflicting approaches of eradicating infection by removing the prosthesis and stabilising fractures by inserting osteosynthetic material into an infected area simultaneously. A two-stage revision is recommended with immediate fracture stabilisation following the removal of the infected components and replacement of a long femoral stem spacer between stages to ensure success. Reimplantation depends on the extent to which infection parameters have decreased and on the ability of the patient to mobilise.

Based on the Muller *et al* study, 87,5% of cases with PJI combined with PPF were successfully treated with fracture stabilisation via plates, intramedullary rods, or cerclages with additional spacers^13^. In this study, with the long femoral stem spacer, 91.67% of cases with PJI combined with PPF were successfully treated. The spacer in this study was created intra-operatively by a self-designed mold. It results in the possibility of making a flexible choice between the types of antibiotics based on what pathogens are involved and their antibiogram^14^. In those cases where multiple pathogens or multi-resistant antibiotics were present, the flexible choice of antibiotics played a crucial role.

We may also perform a bone graft if the PPF has not healed but the PJI has been eradicated. Since the patient is young, if the prosthesis is replaced with a kind of revision prosthesis, it will be difficult for the patient to replace it after many years. Also, some patients can't afford a revision prosthesis (Fig. 4).

According to our study, it must be understood that in the treatment of PJI combined with PPF, if the key to successful treatment of PPF is the long femoral stem spacer, then the key to successful treatment of PJI is debridement and antibiotics. A single pack of bone cement was mixed with 2g of Vancomycin and 2g of Meropenem for six cases with multiple organisms, which resulted in satisfactory results^15,16^. We continued to administer oral antibiotics to the patient for another 4-6 weeks, depending on their antibiogram^17^.

This study has several limitations, including a relatively small number of cases. Despite this, the combination of PJI and PPF is extremely rare^13,18-20^. A literature search was conducted using the search terms "periprosthetic joint infection" and "periprosthetic hip fracture" and very few studies addressed this issue, only five studies with a very small number of patients. Some cases may continue to follow-up after more than two years, and the re-infection rate is very low with an average follow-up time of 27.92 months following a two-stage revision (range 8 to 65 months). The second issue is that we have not yet been able to overcome the anti-rotation problem of the distal femur in our study. The selection of the appropriate size for each patient contributes to minimising the rotation of the distal femur but does not eliminate it. Thirdly, if the periprosthetic fracture is complicated, consisting of numerous bone fragments, our plan will not be suitable because it does not guarantee stability. As a result, it would be more appropriate in this situation to use an external fixation.

## Conclusion

In challenging reconstructive scenarios, we developed a technique that provides mobility, local antibiotic delivery, maintains leg length, and fixes fractures of the bone. The PJI combined with PPF can be treated simultaneously using long femoral stem spacers during the same procedure. This study used an intra-operative mold to create a spacer that allowed for flexible antibiotic selection based on the pathogens and their antibiograms. The results of this study are encouraging, even though long-term data are required to determine its longevity and efficacy.

## References

[1] Brady OH, Garbuz DS, Masri BA, Duncan CP (2000). The reliability and validity of the Vancouver classification of femoral fractures after hip replacement.. J Arthroplasty..

[2] Duncan CP, Haddad FS (2014). The Unified Classification System (UCS): improving our understanding of periprosthetic fractures.. Bone Joint J..

[3] Parvizi J, Alijanipour P, Barberi EF, Hickok NJ, Phillips KS, Shapiro IM (2015). Novel developments in the prevention, diagnosis, and treatment of periprosthetic joint infections.. J Am Acad Orthop Surg..

[4] Ricioli W Jr, Queiroz MC, Guimarães RP, Honda EK, Polesello G, Fucs PM (2015). Prevalence and risk factors for intra-operative periprosthetic fractures in one thousand eight hundred and seventy two patients undergoing total hip arthroplasty: a cross-sectional study.. Int Orthop..

[5] Gehrke T, Alijanipour P, Parvizi J (2015). The management of an infected total knee arthroplasty.. Bone Joint J..

[6] Chen AF, Heller S, Parvizi J (2014). Prosthetic joint infections.. Surg Clin North Am..

[7] Fink B, Fuerst M, Singer J (2005). Periprosthetic fractures of the femur associated with hip arthroplasty.. Arch Orthop Trauma Surg..

[8] Sherman SL, Cunneen KP, Walcott-Sapp S, Brause B, Westrich GH (2008). Custom total femur spacer and second-stage total femur arthroplasty as a novel approach to infection and periprosthetic fracture.. J Arthroplasty..

[9] Liporace FA, Yoon RS, Frank MA, Gaines RJ, Maurer JP, Polishchuk DL (2012). Use of an "antibiotic plate" for infected periprosthetic fracture in total hip arthroplasty.. J Orthop Trauma..

[10] Schwab JH, Pagnano M, Haidukewych GJ, Trousdale R (2003). A technique for treating periprosthetic fractures of the femur associated with deep prosthetic infection.. J Arthroplasty..

[11] Hofmann AA (1999). Two-stage exchange is better than direct exchange in the infected THA.. Orthopedics..

[12] Moyad TF, Thornhill T, Estok D (2008). Evaluation and management of the infected total hip and knee.. Orthopedics..

[13] Müller M, Winkler T, Märdian S, Trampuz A, Renz N, Perka C (2019). The worst-case scenario: treatment of periprosthetic femoral fracture with coexistent periprosthetic infection-a prospective and consecutive clinical study.. Arch Orthop Trauma Surg..

[14] Van Le T, Duong TB, Hien KQ, Ton QNQ, Huyn T, Binh TP (2023). Two-stage revision for treatment of tuberculous prosthetic hip infection: an outcome analysis.. Eur J Orthop Surg Traumatol..

[15] Wang LH, Feng YD, Zhang XW, Jin L, Zhou FL, Xu GH (2021). Elution and Biomechanical Properties of Meropenem-Loaded Bone Cement.. Orthop Surg..

[16] Baleani M, Persson C, Zolezzi C, Andollina A, Borrelli AM, Tigani D (2008). Biological and biomechanical effects of vancomycin and meropenem in acrylic bone cement.. J Arthroplasty..

[17] Le Vavasseur B, Zeller V (2022). Antibiotic Therapy for Prosthetic Joint Infections: An Overview.. Antibiotics (Basel)..

[18] Johnson JP, Cohen EM, Antoci V (2019). Treatment of a periprosthetic femur fracture around an antibiotic spacer with revision and an antibiotic plate.. Arthroplast Today..

[19] Cabral R (2012). Infection in periprosthetic hip fractures.. Hip Int..

[20] Quayle J, Barakat A, Klasan A, Mittal A, Chan G, Gibbs J (2021). Management of peri-prosthetic joint infection and severe bone loss after total hip arthroplasty using a long-stemmed cemented custom-made articulating spacer (CUMARS).. BMC Musculoskelet Disord..

